# Two state “ON–OFF” NLO switch based on coordination complexes of iron and cobalt containing isomeric ligand: a DFT study†

**DOI:** 10.1039/d2ra03867f

**Published:** 2022-08-16

**Authors:** Tamseela Bibi, Tabish Jadoon, Khurshid Ayub

**Affiliations:** Department of Chemistry, COMSATS University Abbottabad Campus 22060 Pakistan Khurshid@cuiatd.edu.pk +92-992-383441 +92-992-383591; Department of Chemistry, GPGC No. 1 Abbottabad KPK Pakistan

## Abstract

Coordination complexes are interesting materials for nonlinear optical (NLO) applications due to their large hyperpolarizability values. Moreover, switchable NLO response is also important in coordination complexes. Herein, we report two state ON–OFF switchable NLO contrast of coordination complexes of Fe and Co containing isomeric ligands. The optical, UV-visible, and electronic properties besides the “ON–OFF” switching effect are calculated using the CAM-B3LYP/6-31+G (d) method. The NLO responses of ligand–metal isomers are qualitatively evaluated through variation in charge transference (CT) style through TD-DFT. The higher *β*_o_ in each isomeric pair is strongly dependent on the HOMO–LUMO gap. The isomer 4b with lowest HOMO–LUMO gap shows the highest NLO response. The charge transfer pattern in these complexes results in variation of their *β*_o_ values. The notable *β*_o_ contrast of 21.15 in isomeric pairs 3a and 3b makes these complexes a favorable material for genuine NLO switches. Hence, the outcome of the current investigation reveals that these ligand–metal isomeric complexes exhibit a two-state switch “ON–OFF” effect.

## Abstract 2

Coordination complexes are interesting materials for nonlinear optical (NLO) applications due to their large hyperpolarizability values.

## Graphical abstract

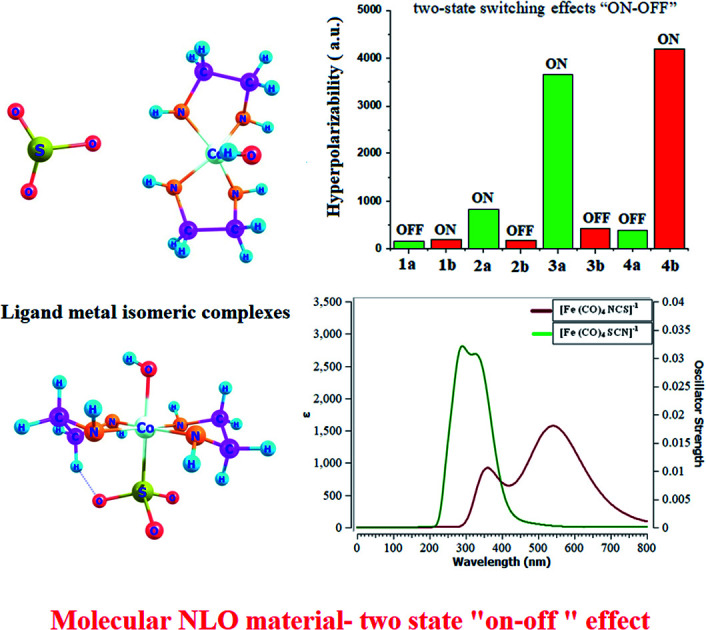

## Introduction

In recent years nonlinear optics (NLO) achieved notable consideration owing to their extensive usage in advanced optoelectronics,^1^ optical switching,^2^ signal processing,^3^ information storage,^4^ and all data processing.^5^ NLO materials generally have properties such as polarization, conjugated structure, phase, amplitude, frequency *etc.* which can be altered for their broad spectrum applications.^6–9^ Amongst NLO materials, switchable NLO molecular materials display a high potential usage in data storage, signal processing and sensing besides optoelectronic technologies. Molecules with switchable second-order nonlinear optical properties have gained a great deal of interest because of their potential use in photonic devices.^10–12^ The progress of innovative nonlinear optical materials is essential for sustainable development in nanoscience and nanotechnology.^13–15^

The literature study revealed that various strategies including bond length alteration, metal–ligand frameworks, excess electron incorporation, extended conjugation in π-linkage, and accommodation of donor–acceptor fragments connected *via* a π-network have been frequently adopted to improve the NLO properties of materials.^16–18^

NLO materials, for instance organic compounds and coordination complexes, have been extensively used in the last few decades.^15,19–24^ Organic compounds are most used as NLO materials due to their ease of synthesis, low cost, and versatility in tailoring their properties. One of the key benefits of using organic materials is that through precise and distinct synthetic modifications of the molecular structures, their physical and chemical properties can be customized.^14,16,25–28^ The assimilation of donor and acceptor fragments connected by employing a π-network proves to be an effective strategy to enhance the NLO properties of organic material. Intramolecular charge transfer (ICT) affects NLO properties that originate primarily from the π-conjugated bridge due to donor to acceptor moieties. The proper acceptor and donor fragments connected using a π-network develop an electron push–pull mechanism that enhances the NLO properties of materials.^20,29–38^ However, the low thermal stability and volatile nature of organic compounds limit their potential use.

Coordination complexes have gained remarkable interest in the field of nonlinear optics due to their more flexible molecular design. The presence of a greater number of diverse charge-transfer transitions of high intensity at quite low energy is tunable by employing the nature, oxidation state, and metal center of the coordination sphere. There exist huge varieties and notably limitless opportunities for metal–ligand combinations that allow tailoring of metal complexes for various potential uses in the switching of NLO response. In these complexes, it is possible to switch molecular architecture, spin state, charge distribution pattern, and magnetic moment.^39^ Mostly 4d and 5d metal complexes exhibit low-energy ligand-to metal charge-transfer (LMCT), metal-to-ligand charge-transfer (MLCT), ligand-to-ligand charge-transfer (LLCT) or intra ligand charge-transfer (ILCT) excitations.^40^ The metal can effectively be the donor or the acceptor moiety or the polarizable bridge of a push–pull system that can act as a dipolar NLO chromophore.^41^ The existence of the metal likewise is a required feature that has been exploited in efficient redox-switching of the NLO properties, which is of great attention in the field of photonics.

The coordination complexes are attractive NLO materials due to optical bands in the visible region and various oxidation states of metal ions. Coordination complexes are useful for optical applications due to the presence of an electron donor and acceptor moieties connected *via* a π-conjugate link.^12,42–44^ Using metal–ligand frameworks in these compounds, the NLO response can be enhanced. This strategy induces metal to ligand charge transfer (MLCT). In coordination complexes, charge transfer excitations occur and they differ in nature either in directions from metal towards ligand, ligand towards metal, or intra ligand.^45–47^ The coordination of the ambidentate ligand with the metal center results in structural isomerism in coordination complexes because the ligand can bind in more than one way to the metal center. The study of various ligand isomers is of potential interest for numerous applications in molecular NLO switches.^12,15,48,49^

Literature reveals that numerous molecular switches have been reported both experimentally and theoretically to investigate their switchable NLO response. The switching NLO properties in the photochromic pair of furylfulgide Aberchrome 540 and dihydrobenzofuran derivatives studied at B3LYP/6-311++G(d,p) level of theory reveal that these systems can be used as efficient molecular NLO switches.^50^ In another theoretical study, photoisomerization effect for dithiazolylarylene was studied.^51^ The outcome of the study demonstrates that dithiazolylarylene is not only a photochromic material but also a reversible NLO switch.^52^ The NLO switching mechanism of the scarce singlet diradical electride molecule has been reported at the CAM-B3LYP/6-311++G (2d,2p) level of DFT.^53^ The easily driven electrons due to the electric field at two opposite ends of the molecule make the material quite suitable for use in molecular NLO-based switch design. Alkalides owing to their large NLO responses exhibit broad applications in electro-optical device fields.^54–56^ The photoactive layer materials were also used to examine the substantial role of graphene quantum dots in NLO-based molecular switches at the B3LYP/6-31G(d) level of DFT.^57–60^

The linkage isomers have been widely investigated. These switchable organometallics have ambidentate ligands, which can have their coordination geometry altered by subjecting the crystal to outside stimuli like heat or light. Photoinduced linkage isomers exist as long-lived metastable states in the crystal (with excited states), and lifetimes that are temperature dependent.^61–63^ A variety of linkage isomers have been studied, including nitrosyl, sulfur dioxide, dinitrogen, and nitrite complexes. Recent advancements in NLO materials development include [Ni(dppe)(NO_2_)Cl], alkali metals doped 2N-atoms functionalized corannulene complexes, 5-phenylamino-isophthalic acid, 9,10-bis(phenylethynyl) anthracene, Zn complexes based on N_2_O_4_-type pro-ligand iridium complexes, Pt(ii) dithienylethene complexes. Sulfur and nitrogen-bonded thiocyanato linkage isomers of dicarbonyl-π-cyclopentadienyl iron and tricarbonyl-π-cyclopentadienyl molybdenum have been investigated for their NLO response.^10–12,16,64^

Therefore, we are interested to explore the NLO effects of ligand isomeric complexes of transition metals with different ambidentate ligands. Due to the nature of charge-transfer excitations, these ligand isomeric complexes are anticipated to exhibit NLO contrasts. We herein, theoretically investigated within the DFT framework the geometric, electronic, and optical properties of ligand–metal isomers of iron and cobalt with different ambidentate ligands.^45^

## Computational methodology

Gaussian 09 program is used for all quantum chemical simulations. Geometries of all complexes are optimized at CAM-B3LYP along with 6-31+G (d) basis set.^65^ CAM-B3LYP is a range separated functional which is selected on account of capturing intra-molecular charge transfer within complexes.^66–70^ To get the most stable spin state, geometry optimization of ligand–metal complexes is performed at various spin states. The most stable structures of ligand–metal isomeric complexes are then presented for the calculations of the HOMO–LUMO gap, dipole moment (*μ*_o_), polarizabilities (*α*_o_) and hyperpolarizability (*β*_o_). All properties are calculated with 6-31+G(d) basis set while using CAM-B3LYP method except hyperpolarizability which is calculated at 6-31+G(d,p) basis set. The mean polarizability (*α*_o_) and hyperpolarizability (*β*_o_) are documented in this manner:1
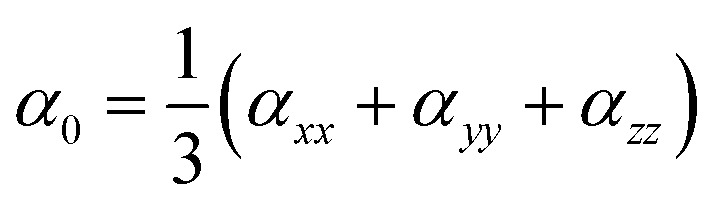
2
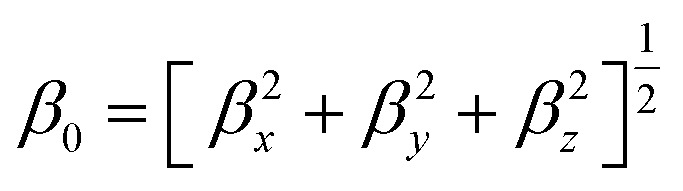
where*β*_*x*_ = *β*_*xxx*_ + *β*_*xyy*_ + *β*_*xzz*_*β*_*y*_ = *β*_*yyy*_ + *β*_*yzz*_ + *β*_*yxx*_and *β*_*z*_ = *β*_*zzz*_ + *β*_*zxx*_ + *β*_*zyy*_

For understanding the large hyperpolarizability value, a two-level model is considered.^71,72^ As stated by the two-level method

3 *β*_o_ ≈ Δ*μ* × *f*_o_/Δ*E*^3^

where, Δ*μ* is variation in dipole moment, *f*_o_ is the oscillator strength and Δ*E* is the transition energy.

According to the relation, hyperpolarizability is related to oscillator strength and variation in *μ* while it is inversely related to the transition energy.

Time-dependent density functional theory (TD-DFT) simulations are conducted by the TD-CAM-B3LYP/6-31+G (d) method to attain dominant excited states, oscillator strength (*f*_o_), absorption maxima and energies needed for excitation of the most stable geometries.

## Results and discussion

Outcomes of frequency simulations affirmed the geometries are products and not transition states.

### Relative energies of ligand–metal isomers

The energy is an imperative stability parameter, complexes with lower energies are more stable than complexes having higher energies. The relative energies relating to the utmost stable complexes are displayed in Table 1.

**Table 1:** Relative energies (kcal mol^−1^) of ligand–metal isomers in various spin states

Ligand–metal isomers		Relative energies		
		Singlet	Triplet	Quintet
1a	Co–ONO	0.5	6.3	0.0
1b	Co–NO_2_	0.0	7.9	2.6
2a	Fe–ON	36.9	6.1	0.0
2b	Fe–NO	0.0	10.1	13.0
3a	Fe–NSC	90.8	77.8	0.0
3b	Fe–SCN	0.0	10.8	55.2
4a	Co–SO_3_	0.0	90.5	90.4
4b	Co–OSO_2_	0.0	43.8	25.1

**Fig. 1:**
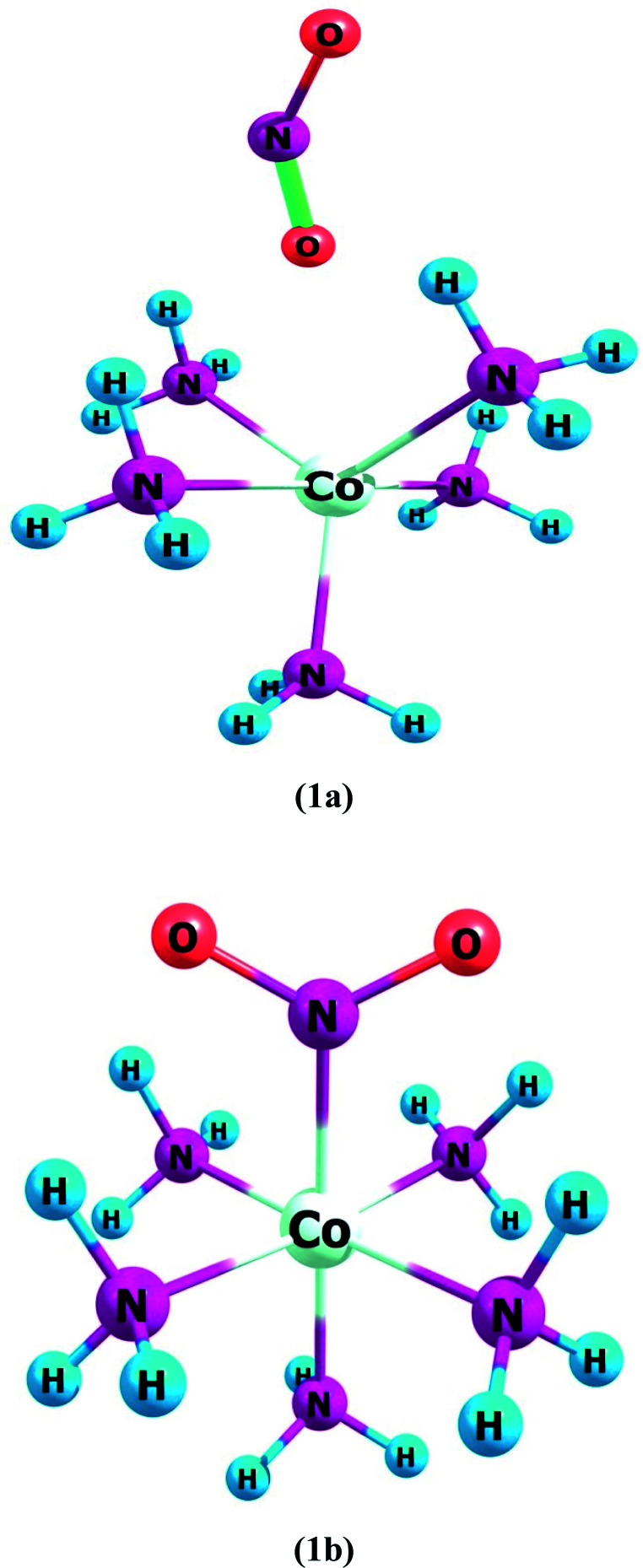
Geometries of ligand–metal isomers (1a) Co–ONO and (1b) Co–NO_2_.

Complex 1a has an ONO ligand (nitrito), which exists in its isomeric NO_2_ in complex 1b. In ligand–metal isomeric complex 1a, the nitrogen atom is bonded with cobalt metal (Fig. 1). Spin multiplicities results reveal that complex 1a is most stable in the quintet state. The quintet spin state of 1a is comparatively higher in stability than its singlet and triplet states by amounts of 0.5 and 6.3 kcal mol^−1^, respectively. Furthermore, the singlet spin state of 1b, is comparatively lower in energy compared to its triplet and quintet states by 7.9 and 2.6 kcal mol^−1^, respectively.

**Fig. 2:**
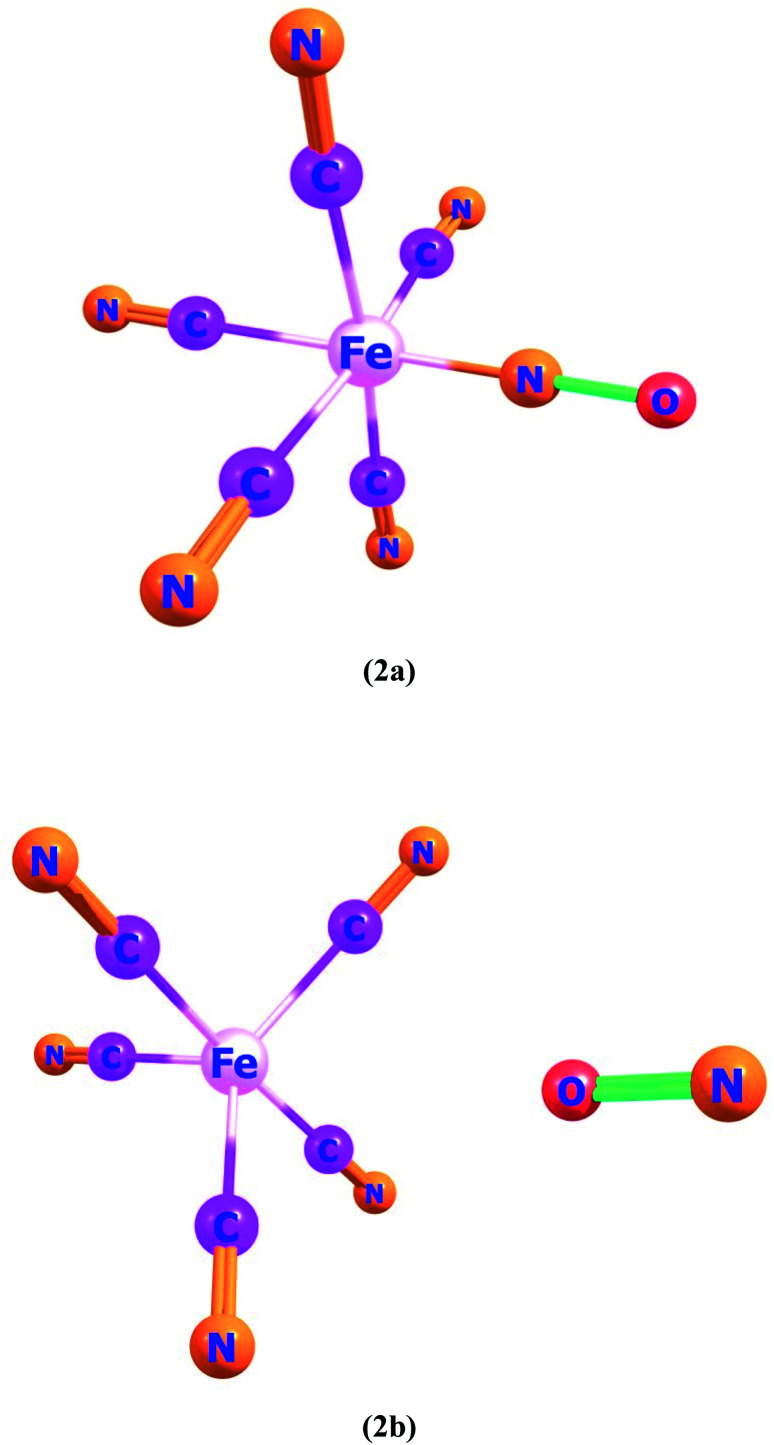
Geometries of ligand–metal isomers (2a) Fe–ON and (2b) Fe–NO.

Ligand–metal complexes 2a and 2b contain iron with nitric oxide ambidentate ligand. In complex 2a and 2b, oxygen and nitrogen atoms of NO are bonded with iron metal, respectively (Fig. 2). The most stable spin state of complex 2a is quintet whereas complex 2b is a singlet. The quintet spin state of 2a stands more stable than its singlet and triplet states by 36.9 and 6.1 kcal mol^−1^, correspondingly. The most stable spin state (singlet) of complex 2b, is almost 10.1 and 13.0 kcal mol^−1^, lower in energy than triplet and quintet states, respectively because of the strong field NO ligand.

**Fig. 3:**
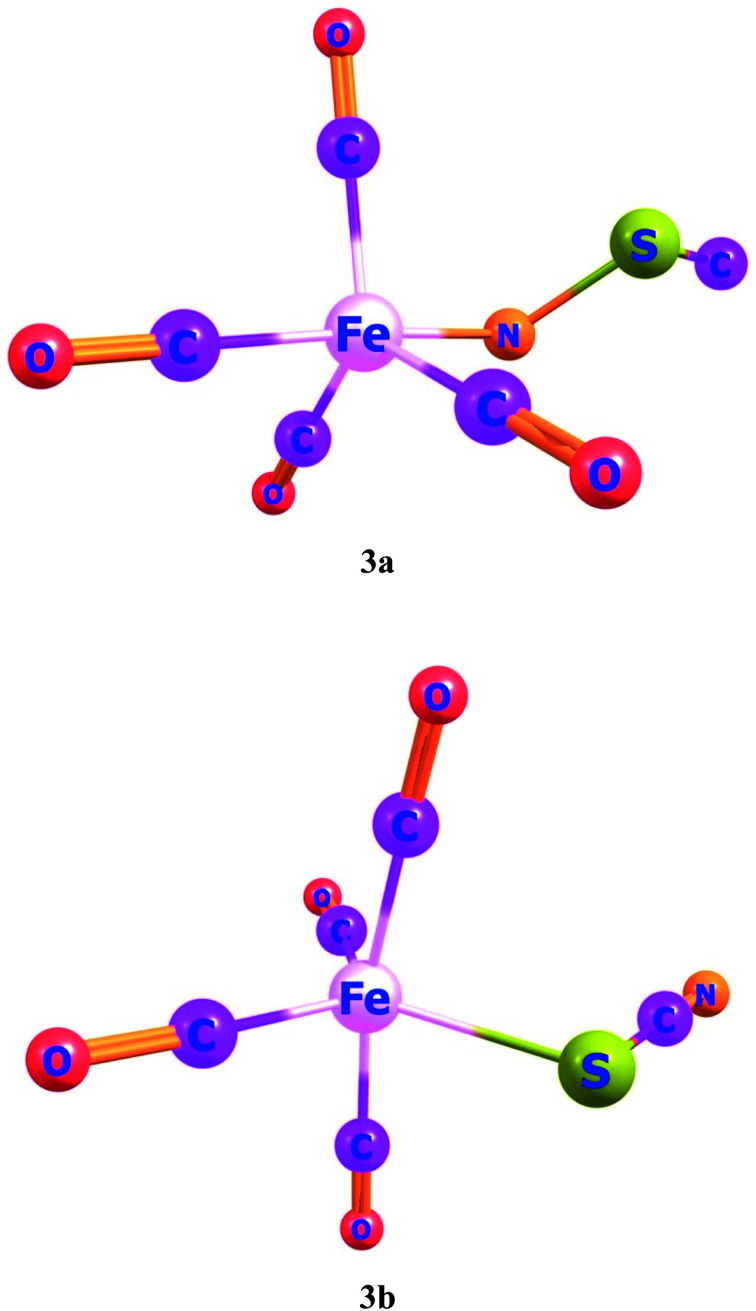
Geometries of ligand–metal isomers (3a) Fe–NSC and (3b) Fe–SCN.

Ligand–metal isomeric complexes 3a and 3b, consist of two linkage isomers of thiocyanate ambidentate ligand. In complex 3a, the nitrogen atom of a ligand is bonded with iron whereas, in complex 3b, the sulfur atom is bonded with iron (Fig. 3). Complex 3a is more stable in the quintet spin state. The singlet and quintet states of 3a, are about 90.8 and 77.8 kcal mol^−1^, greater in energy than its triplet state. The S-bonded complex 3b is more stable in the singlet spin state, which is about 10.8 and 55.2 kcal mol^−1^, lower in energy compared to its triplet and quintet spin states, respectively.

**Fig. 4:**
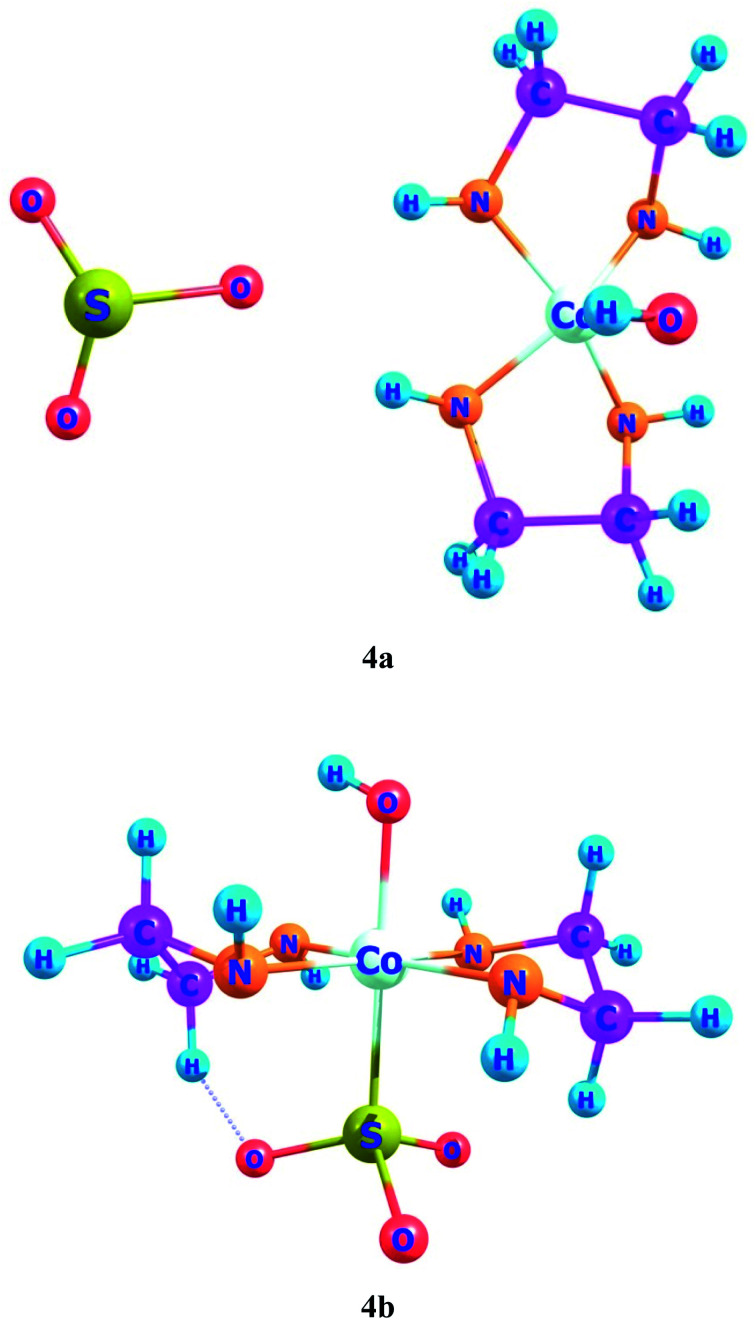
Geometries of ligand–metal isomers (4a) Co–SO_3_ and (4b) Co–OSO_2_.

In ligand–metal, isomeric complexes 4a and 4b, sulfur and oxygen atoms of ligand are bonded with cobalt metal (Fig. 4). Both 4a and 4b complexes remain more stable in the singlet states. The singlet state of 4a is rather more stable as compared to its singlet and quintet states by 90.5 and 90.4 kcal mol^−1^, respectively. The results of relative energies of most stable spin states reveal that the singlet state of 4b, shows higher stability than triplet and quintet spin states. 4b's triplet and quintet states exhibit energies of 43.8 and 25.1 kcal mol^−1^, correspondingly, which become higher than its lowest energy singlet state. The OSO_2_ is a strong field ligand therefore, it pairs up electrons in complex and gives stable geometry in a singlet spin state.

### Electronic properties of ligand–metal isomers

FMO's investigation is carried out to get evidence concerning energies of occupied (HOMO) and virtual orbitals (LUMO) of ligand–metal isomers. Reactivity of the system is inspected by variations in energies of HOMO and LUMO. The reactivity and stability of a species are characterized by a HOMO–LUMO gap. The species with smaller H–L gap show high reactivity, and high conductivity and are generally less stable. A small Δ*E* value is a distinctive feature of less stable materials although easily polarizable suggesting simpler electronic transition and, therefore, improved NLO properties of the complexes.

**Fig. 5:**
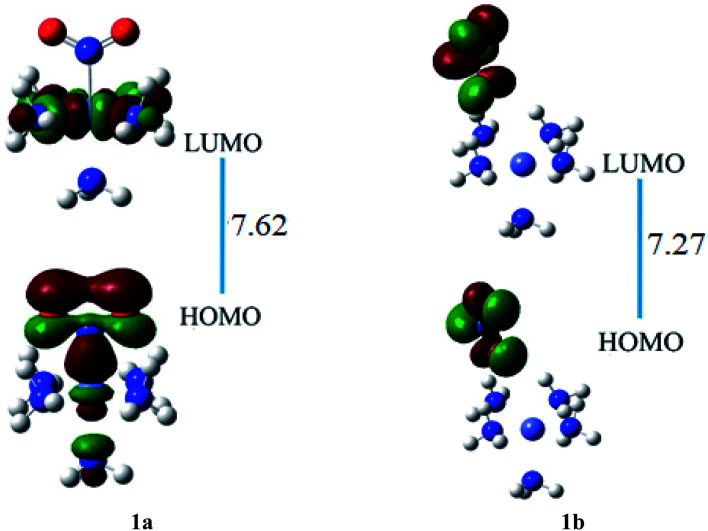
The HOMO and LUMO visualization of ligand–metal isomers 1a and 1b.

The results of the FMO investigation are given in Table 2. The HOMO of ligand–metal isomer 1a appeared at −16.96 while its LUMO at −9.34 eV beside H–L gap of 7.62 eV. The HOMO and LUMO energies of ligand–metal isomer 1b are 16.49 and 9.21 eV, respectively, with a 7.27 eV H–L energy gap. The H–L gap of 1a (in which oxygen is bonded to Fe) is higher compared to the H–L gap of 1b (in which nitrogen is bonded to Fe) due to efficient orbital hybridization in the latter (Fig. 5).

**Table 2:** FMO and UV-Vis spectroscopic outcomes of ligand–metal isomers

Ligand–metal isomers	*E*_HOMO_ (eV)	*E*_LUMO_ (eV)	H–L gap (eV)	*E*_excitation_ (eV)	*f*_o_ (a.u.)	*λ*_max_ (nm)
1a	−16.96	−9.34	7.62	3.26	0.01	256
1b	−16.49	−9.21	7.27	4.82	0.23	380
2a	−11.18	−5.26	5.92	3.73	0.03	332
2b	−2.71	4.54	7.26	3.97	0.02	311
3a	−4.26	2.08	6.34	4.96	0.01	249
3b	−3.84	2.61	6.46	2.27	1.03	545
4a	−11.99	−5.21	6.77	2.47	0.01	500
4b	−7.62	−3.78	3.84	2.79	0.01	444

**Fig. 6:**
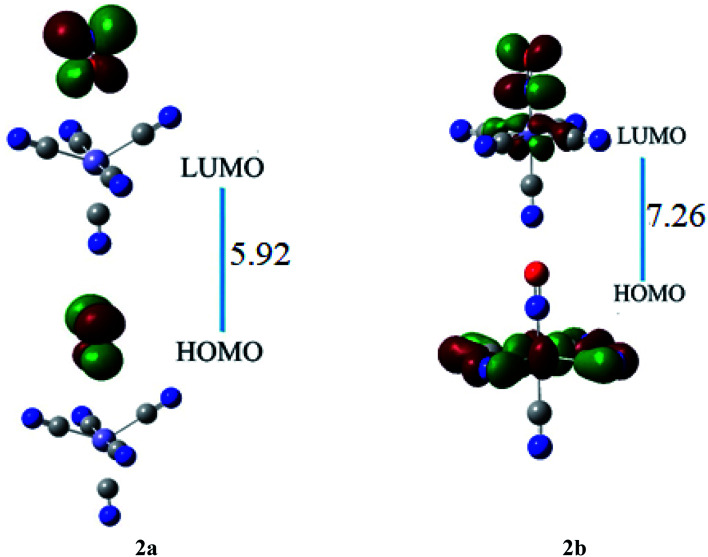
The HOMO and LUMO visualization of ligand–metal isomers 2a and 2b.

The HOMO of ligand–metal isomer 2a, appeared at −11.18 whereas its LUMO was at −5.26 eV, beside the H–L energy gap of 5.92 eV. The HOMO and LUMO of ligand–metal isomer 2b, exist at −2.71 and 4.54 eV, correspondingly beside 7.26 eV of the H–L gap (Fig. 6). The lower H–L gap in 2a, is owing to greater orbital hybridization between the highly electronegative oxygen atom and iron centre.

**Fig. 7:**
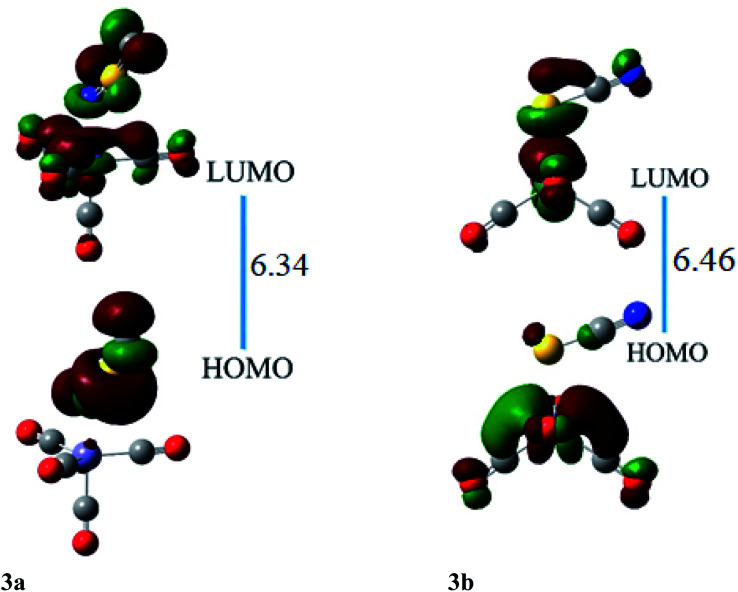
The HOMO and LUMO visualization of ligand–metal isomers 3a and 3b.

HOMO and LUMO are located at 4.26 and 2.08 eV, respectively, besides the H–L gap of 6.34 eV in ligand–metal isomer 3a (Fig. 7). The HOMO of 3b is situated at −3.84 eV while its LUMO at 2.61 eV. The H–L gap in 3b (sulfur bonded isomer) is 6.46 eV, which is higher compared to the H–L gap of isomer 3a (nitrogen bonded isomer). The reduced H–L gap of 3a is because of the high electronegativity of the N atom of NSC ligand bonded with iron metal which causes efficient orbital hybridization.

The HOMO of ligand–metal isomer 4a is located at −11.99 whereas its LUMO is present at −5.21 eV beside H–L gap of 6.77 eV in the complex. The H–L gap in 4b is 3.84 eV which is found to be the lowest among all studied complexes. The HOMO of the 4b is located at −7.62 eV, whereas LUMO is at −3.78 eV. The substantial decrease in H–L gap in 4b compared to 4a is due to the effective orbital overlap between the highly electronegative oxygen atom of the ligand and cobalt metal center. Moreover, the results of FMO reveal that a noteworthy drop in the H–L gap is noticed in isomeric complexes where a highly electronegative atom of ligand bonded with an electropositive metal center.

### Nonlinear optical properties of ligand–metal isomers

The transference of charge both from metal to ligand and from ligand to metal assures the actual NLO response of ligand–metal isomers. These complexes contain functioning centers of an additional electron that certainly be transported to an electron-scarce place. This intramolecular charge transference assures the advancement of NLO properties of ligand–metal isomers. The outcomes of the NLO investigation are listed in Table 3.

**Table 3:** Dipole moment (*μ*_o_), mean polarizabilities (*α*_o_), mean first hyperpolarizabilities (*β*_o_), and ratios of hyperpolarizabilities of ligand–metal isomers calculated at CAM-B3LYP/6-31G+(d,p) level

Complexes	*μ*_o_ (Debye)	*α*_o_ (a.u.)	*β*_o_ (a.u.)	State	Ratio (ON/OFF)
1a	7.82	83	158	OFF	
1b	10.61	88	192	ON	1.21
2a	5.48	138	828	ON	4.78
2b	7.23	137	173	OFF	
3a	5.35	165	3662	ON	21.15
3b	5.94	140	431	OFF	
4a	11.67	134	390	OFF	
4b	2.10	165	4184	ON	10.71

Ligand isomers complexes consist of ambidentate ligand which contains two different donor atoms of different electronegativities. As a result of the difference in electronegativities of donor atoms of ligand, the shift of electronic density is different in these isomeric complexes. Because of the different patterns of electronic densities distribution, these isomers show a contrast in NLO response.

Complex 1, comprises two isomers (1a & 1b) of NO_2_ ambidentate ligand. In isomer 1a, cobalt is attached to the oxygen atom of the ligand, and in 1b, the nitrogen atom of the ligand is attached to the Cobalt center (Fig. 1).

These two isomers have different hyperpolarizability values. The *β*_o_ of 1a is 158 a.u. while that of 1b is 192 a.u. The *β*_o_ of isomer 1b, is 1.21 times higher compared to 1a, due to the charge transference pattern and high oscillator strength. The greater *β*_o_ of 1b is consistent with its small H–L gap than 1a (*vide supra*). In isomer 1a, HOMO density is mainly situated equally on metal and ligand, though LUMO density is only sited on metal. This demonstrates the transfer of electronic cloud from ligand to metal on excitation from HOMO to LUMO and hence called MLCT. In isomer 1b, HOMO and LUMO densities are equally situated on the ligand moiety. In ligand isomer 1b, the orbital overlap is inadequate to develop an effectual electronic densities distribution. The intra-ligand transference of charge transitions in 1b, is termed ILCT. The high energy of transition beside poor distribution of charge and lower dipole moment are observed in 1a. The donor and acceptor features between molecule may influence the NLO response and thus charge transference cause variation in the *β*_o_ value of these ligand–metal isomers.

The *β*_o_ values of ligand–metal isomeric complexes 2a and 2b are 828 and 173 a.u., respectively. The *β*_o_ of 2a stands 4.78 times higher in value than its isomer 2b. The HOMO and LUMO densities in 2a, are equally present on the ligand. The intra-ligand charge transference mode (ILCT) of 2a, is displayed in Fig. 6. In 2b, the HOMO density is placed at the metal center, however, the LUMO density is situated at ligand as well on metal sites of the complex (MLCT). In isomer 2a, where iron is bonded with the oxygen atom of ligand, the electronic density is shifted more towards ligand than in isomer 2b (where Fe is bonded with the nitrogen atom of ligand). The greater shifting of electronic density towards ligand in isomer 2a is due to the higher electronegativity of the oxygen atom. The greater *β*_o_ of isomer 2a, compared to 2b, is consistent with its lesser transition energies and reduced H–L gap besides bathochromic effects.

The *β*_o_ of ligand–metal isomer 3a is 3662 a.u., however that of 3b is 431 a.u. The *β*_o_ of isomer 3a, is 21.15 times higher in value compared to 3b. The greater contrast of *β*_o_ values is observed in 3a and 3b, among all studied isomeric complexes. The visualization of HOMO and LUMO densities of 3a and 3b are presented in Fig. 7. The results of charge transference excitation in isomer 3a, show that HOMO density is entirely situated at the ligand site while LUMO densities are equally at ligand and metal moieties. The spreading of HOMO and LUMO densities in this isomeric pair suggests ligand to metal charge transfer (LMCT).

In 3b, the density of HOMO is present at the metal part while LUMO density is situated at both metal and ligand (MLCT). The larger hyperpolarizability of 3b than 3a is due to efficient charge transference from Fe to the ligand in the complex.

**Fig. 8:**
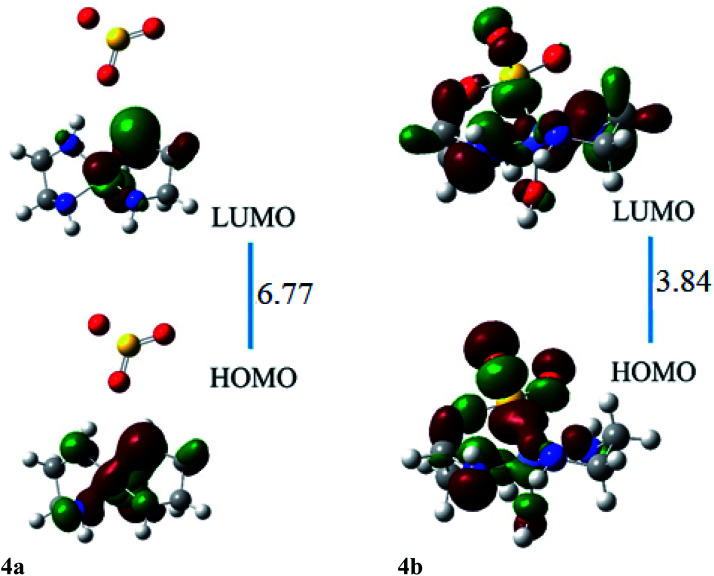
The HOMO and LUMO visualization of ligand–metal isomers 4a and 4b.

The ligand–metal isomers, 4a & 4b have hyperpolarizabilities of 390 a.u. and 4184 a.u., respectively. Isomer 4b, exhibit a high *β*_o_ compared to 4a, beside its lesser H–L gap. The HOMO and LUMO densities are equally spread over metal in isomer 4a (Fig. 8). The orbital overlap is insufficient to cause an efficient distribution of electronic density in 4a, due to the low electronegativity of the S atom in the ligand. In ligand–metal isomer 4b, HOMO density is situated at metal and ligand parts. Likewise, LUMO density is also situated equally at ligand and Co moieties in isomer 4b where charge transfer from ligand to metal is noticed (MLCT). The higher electronegativity of the oxygen atom of ligand bonded with cobalt metal in isomer 4b, causes effective charge distribution which results in its higher *β*_o_. The noteworthy intra-molecular transference of charge in ligand–metal isomers, declares their potential usage, as distinctive NLO materials for numerous applications. Analysis of the results in Tables 2 and 3 reveals that the H–L gap is the main deciding factor for hyperpolarizabilities. The lower the HOMO–LUMO gap, the higher is the hyperpolarizability. This statement is valid for all complexes.

### Molecular absorption spectra of ligand–metal isomers

**Fig. 9:**
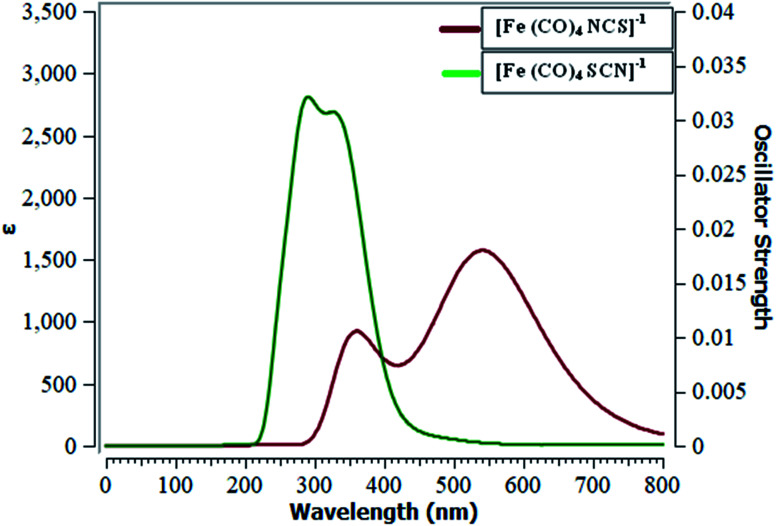
UV-visible spectra of metal isomers of SCN.

TD-DFT is a reliable method to develop ultraviolet-visible (UV-visible) absorption spectra. Ligand–metal isomers show considerable absorption in the entire ultraviolet and visible region to some extent. Results of excited state calculations of ligand–metal complexes are displayed in Table 2. The UV-visible spectra of isomers 3a & 3b are presented in Fig. 9, however, all remaining complexes are given in ESI.† We herein, calculated twenty electronic states to attain vital excited states. The results reveal that ligand–metal isomers 1a and 1b, show maximum absorption (*λ*_max_) at 256 and 380 nm, respectively. The bathochromic shifting of absorption maximum is observed in 1b compared to 1a. The oscillator strength (*f*_o_) or the probability of absorption for electronic transition in 1a and 1b, are 0.01 and 0.23 a.u. respectively. The *λ*_max_ of isomer 2a is moved to a longer wavelength compared to the *λ*_max_ of isomer 2b. The oscillator strength of isomers 2a and 2b, are 0.03 and 0.02 a.u. respectively. The red shifting of *λ*_max_ in 2a, compared to its isomer 2b, is consistent with its higher hyperpolarizability and lower excitation energy.

The isomers 3a & 3b, show absorption maxima at 249 and 545 nm, respectively. Isomers 3a and 3b, show oscillator strength of 0.01 and 1.03 a.u. respectively. *λ*_max_ of 3b, is red shifted regarding 3a, which matches with its lower excitation energy. The electronic transitions of ligand isomers 4a and 4b, appeared at 500 and 444 nm, respectively. The oscillator strength in both isomers is 0.01 a.u. Isomer 4a has lower excitation energy than corresponding isomer 4b, which is associated with its larger bathochromic shift of *λ*_max_. The predictions of UV-visible absorption spectral results we obtained reflect better NLO properties of ligand–metal isomers.

### Ligand–metal isomeric NLO switches

**Fig. 10:**
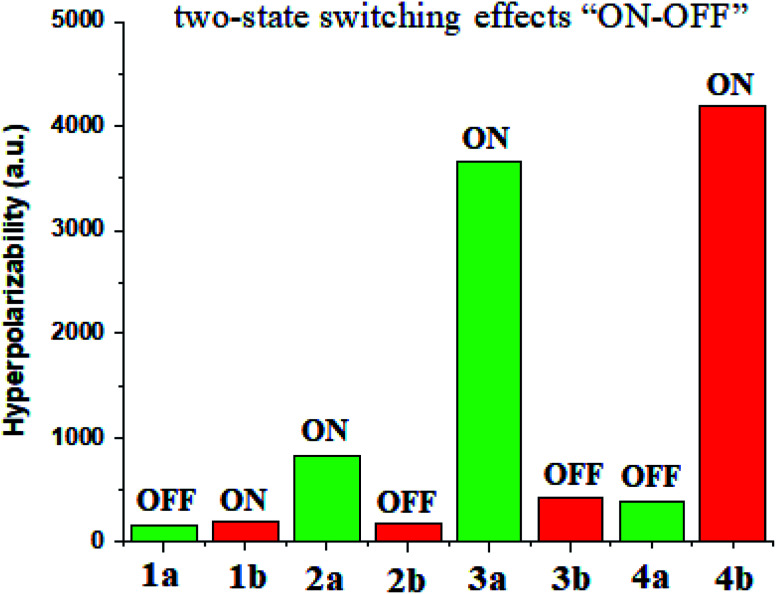
Switch ON–OFF ratios of ligand–metal isomers.

Materials having the contrast of NLO properties are of significant attention owing to their extensive use in many devices. In the current work, we investigated the NLO switching effects of ligand–metal isomeric complexes. We here focused on the extent of hyperpolarizability values of these complexes besides their switching ratios (Fig. 10).

Ligand–metal isomer 1b shows a *β*_o_ value which is 1.21 times more than 1a. Therefore, isomer 1b is switched “ON” state while 1a is switched “OFF” state. Likewise, isomer 2a shows 4.78 times more *β*_o_ than the corresponding 2b isomer. Ligand–metal isomer 2a is examined as switch “ON” whereas, 2b as switch “OFF” state. The *β*_o_ of 3a, is 21.15 times greater than its isomeric complex 3b. Isomer 3a with a greater *β*_o_ value than 3b, is termed as switch “ON” state but 3b as switch “OFF” state. The highest contrast in *β*_o_ values is observed in isomeric pair 3a and 3b. Also, ligand–metal isomer 4b, with 10.71 times larger *β*_o_ value than 4a, is switch “ON” state whereas 4a is switch “OFF” state. Hence, the results reveal that these ligand–metal isomeric pairs show two-state switching effects “ON–OFF”.

## Conclusions

DFT study is performed to investigate the relative stability, molecular absorption spectra, and NLO responses besides switching effects of ligand–metal isomeric complexes. Larger contrast in *β*_o_ values of studied complexes ensure their potential use as a switchable NLO material. The outcomes of excited state investigations show that electronic transitions and charge density distributions *via* MLCT, LMCT and ILCT, mark the difference in NLO behavior in these isomers. Transition energies and H–L gaps of these isomeric pairs mainly contribute to the comparative variations of *β*_o_ values. Larger contrast of the *β*_o_ values has been observed in these ligand–metal isomeric complexes. The highest NLO contrast in *β*_o_ values is seen in 3a and 3b, isomeric pairs. The highest *β*_o_ value of 4184 a.u. is seen in 4b, that reflects its strong NLO properties. Hopefully, coordination complexes of Fe and Co with isomeric ligands are talented to become redox-triggered switchable NLO materials for the development of NLO devices.

## Funding declaration

The authors admit technical and financial support from CUI, Abbottabad Campus, & HEC Pakistan (Grant No. 1899, 2469, 2981).

## Conflicts of interest

The authors declare that they have no known competing financial interests or personal relationships that could have appeared to influence the work reported in this paper.

## Supplementary Material

RA-012-D2RA03867F-s001
