# Supplementary material for: Two state “ON–OFF” NLO switch based on coordination complexes of iron and cobalt containing isomeric ligand: a DFT study
Source: RSC Adv. 2022 Aug 16;12(36):23204–14. doi: 10.1039/d2ra03867f (PMC9380411; doi:10.1039/d2ra03867f)
Supplement: RA-012-D2RA03867F-s001 [file RA-012-D2RA03867F-s001.pdf]

Electronic Supplementary Material (ESI) for RSC Advances.
This journal is © The Royal Society of Chemistry 2022

Two state "ON-OFF" NLO switch based on coordination complexes of iron and cobalt containing isomeric ligand: A DFT study

Tamseela Bibi[a], Tabish Jadoon[a,b], Khurshid Ayub[a]

a Department of Chemistry, COMSATS University Abbottabad Campus, 22060, Pakistan
b Department of Chemistry, GPGC No.1 Abbottabad, KPK, Pakistan

Corresponding Author

Khurshid Ayub

Tel: +92-992-383591

Fax: +92-992-383441

Email: Khurshid@cuiatd.edu.pk

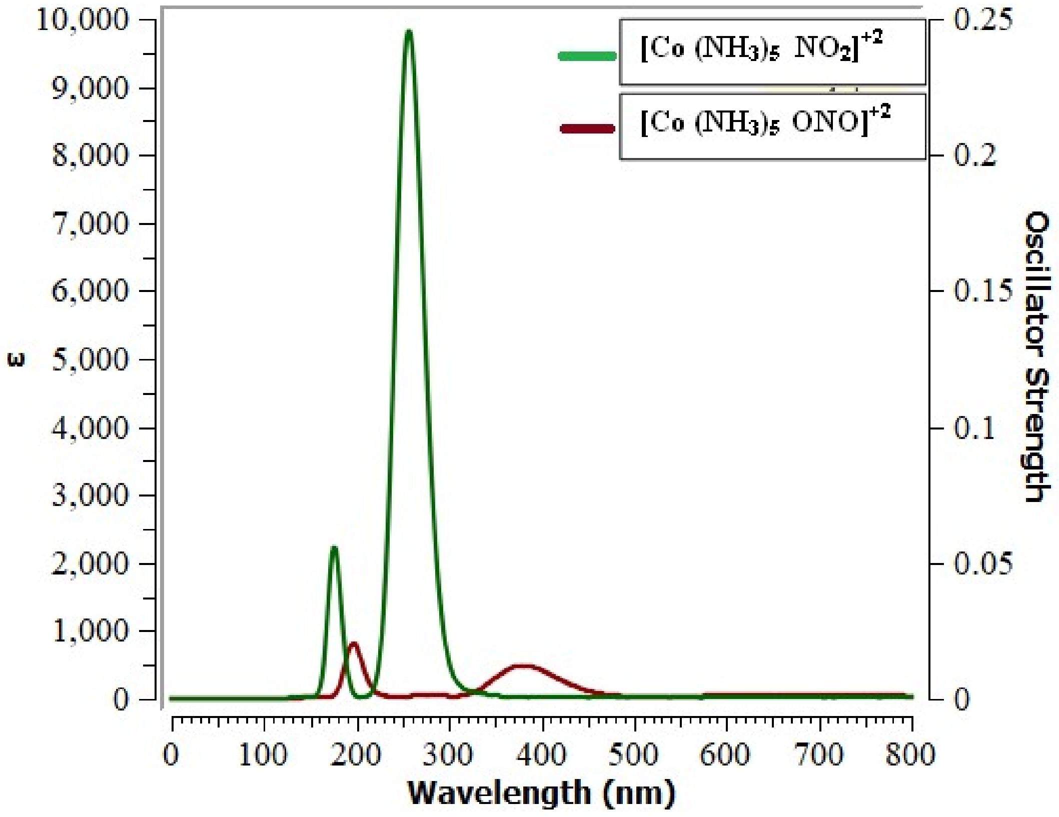

Figure S1: UV-Visible absorption spectra of ligand complexes of NO_2

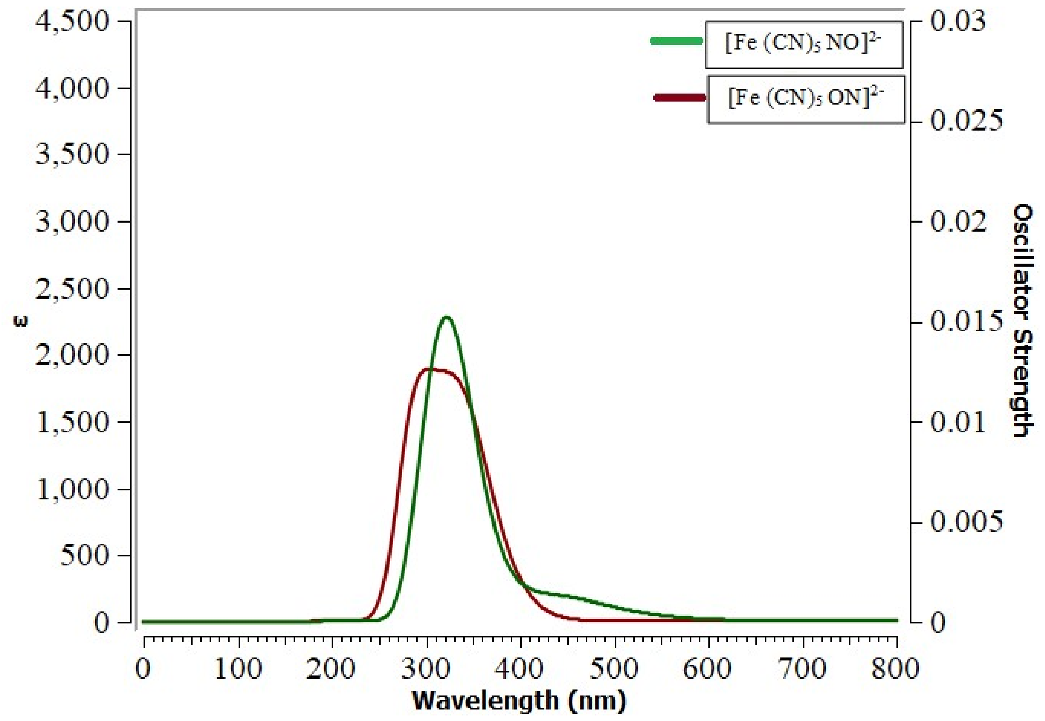

Figure S2: UV-Visible absorption spectra of ligand complexes of NO

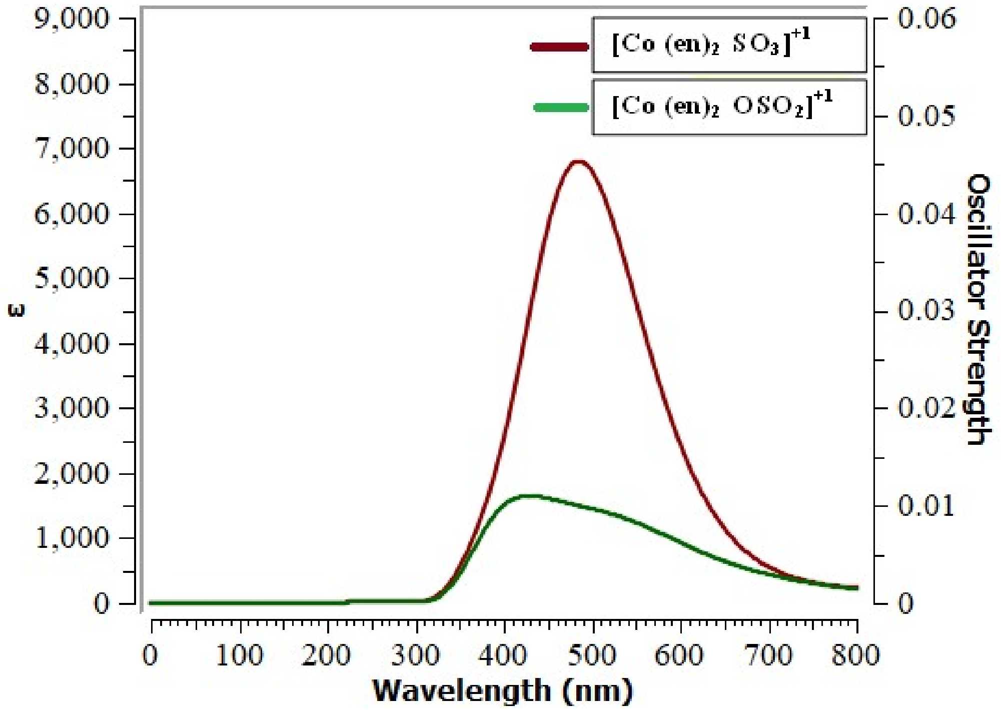

Figure S3: UV-Visible absorption spectra of ligand complexes of SO_3
